# A framework for managing infectious diseases in rural areas in low- and middle-income countries in the face of climate change—East Africa as a case study

**DOI:** 10.1371/journal.pgph.0003892

**Published:** 2025-01-30

**Authors:** Katherine E. L. Worsley-Tonks, Shaleen Angwenyi, Colin Carlson, Guéladio Cissé, Sharon L. Deem, Adam W. Ferguson, Eric M. Fèvre, Esther G. Kimaro, David W. Kimiti, Dino J. Martins, Lutz Merbold, Anne Mottet, Suzan Murray, Mathew Muturi, Teddie M. Potter, Shailendra Prasad, Hannah Wild, James M. Hassell

**Affiliations:** 1 Lyssavirus Epidemiology and Neuropathology Unit, Institut Pasteur, Paris, France; 2 Global Health Program, Smithsonian Conservation Biology Institute, Washington, DC, United States of America; 3 International Livestock Research Institute, Nairobi, Kenya; 4 Department of Epidemiology of Microbial Diseases, Yale University School of Public Health, New Haven, Connecticut, United State of America; 5 Department of Epidemiology and Public Health, Swiss Tropical and Public Health Institute, Basel, Switzerland; 6 Faculty of Science, University of Basel, Basel, Switzerland; 7 Institute for Conservation Medicine, Saint Louis Zoo, Saint Louis, Missouri, United States of America; 8 Gantz Family Collection Center, Field Museum of Natural History, Chicago, Illinois, United States of America; 9 Institute of Infection, Veterinary and Ecological Sciences, University of Liverpool, Liverpool, United Kingdom; 10 Nelson Mandela African Institution of Science and Technology, Arusha, Tanzania; 11 Grevy’s Zebra Trust, Nairobi, Kenya; 12 Turkana Basin Institute, Stony Brook University, Stony Brook, New York, United States of America; 13 Mazingira Centre, International Livestock Research Institute, Nairobi, Kenya; 14 Integrative Agroecology Group, Research Division Agroecology and Environment, Agroscope, Zurich, Switzerland; 15 International Fund for Agricultural Development; Sustainable Production, Markets and Institutions Division, Rome, Italy,; 16 Kenya Zoonotic Disease Unit, Nairobi, Kenya; 17 Department of Veterinary Medicine, Dahlem Research School of Biomedical Sciences (DRS), Freie Universität Berlin, Berlin, Germany; 18 School of Nursing, University of Minnesota, Minneapolis, Minnesota, United States of America; 19 Center for Global Health and Social Responsibility, University of Minnesota, Minneapolis, Minnesota, United States of America; 20 Department of Surgery, University of Washington, Seattle, Washington, United States of America; Monash University, AUSTRALIA

## Abstract

Climate change is having unprecedented impacts on human health, including increasing infectious disease risk. Despite this, health systems across the world are currently not prepared for novel disease scenarios anticipated with climate change. While the need for health systems to develop climate change adaptation strategies has been stressed in the past, there is no clear consensus on how this can be achieved, especially in rural areas in low- and middle-income countries that experience high disease burdens and climate change impacts simultaneously. Here, we highlight the need to put health systems in the context of climate change and demonstrate how this can be achieved by taking into account all aspects of infectious disease risk (i.e., pathogen hazards, and exposure and vulnerability to these pathogen hazards). The framework focuses on rural communities in East Africa since communities in this region experience climate change impacts, present specific vulnerabilities and exposure to climate-related hazards, and have regular exposure to a high burden of infectious diseases. Implementing the outlined approach can help make health systems climate adapted and avoid slowing momentum towards achieving global health grand challenge targets.

## 1. Introduction

**Climate change** (see Glossary of terms in S1 Text) is one of the greatest global health challenges of the twenty first century [1–3]. Hotter global temperatures, more severe storms, rising oceans, and extreme droughts are leading to population displacement, local and international conflict, food and water insecurity, disrupted global trade, rising household costs, and collapse of social infrastructure and stability [1,4]. While different regions of the world experience different types of climate change impacts and associated social, economic, and environmental consequences, all regions experience unprecedented climatic extremes, and all suffer direct or indirect adverse human health impacts [3].

Direct and indirect health impacts of climate change, include non-communicable diseases such as heat stress, cardiovascular diseases, nutritional deficiencies, mental and/or physical stress, and exposure to pollution [5]. These health impacts are a consequence of climate-related hazards (e.g., high temperatures, flooding) which can individually or combined (compounding or cascading) increase **disease risk** [1,6,7]. For example, extreme heat has been linked to 98 million more people globally reporting moderate to severe food insecurity in 2020 than annually in 1980–2010 [4], reversing progress towards achieving Sustainable Development Goals such as hunger eradication [1,4].

Infectious diseases are another important health risk linked to climate change [4,5,8], and the pathogens associated with these infectious diseases can be considered as hazards [9,10], which will now be referred to as **pathogen hazards**. Global changes in climate and weather conditions are causing a shift in vector (e.g., ticks and mosquitoes) and **wildlife reservoir** distributions, altering the distribution and evolution of known and novel pathogens [11], potentially increasing spillover risk to humans [12]. Additionally, infectious diseases that are not directly impacted by climate change are increasingly recognized as being impacted indirectly, which is the case for several neglected tropical diseases (NTDs) in low- and middle-income countries (LMICs) (e.g., leptospirosis, echinococcosis) [13–16].

While pathogen hazards are of increasing concern with climate change, an increase in these health hazards alone does not necessarily equate to an increase in disease risk (likelihood of succumbing to disease) [9,10]; individual **exposure** and **vulnerability** also play a role [9,17,18]. Differences in exposure are linked to differences in human behavior such as socio-economic status or practices which can influence exposure to pathogen hazards. Vulnerability can be at the individual level, with variation in immunity and therefore susceptibility to infections, or at the population level, with differences in access to healthcare. People who are most vulnerable to pathogen hazards and have regular exposure to pathogen hazards have a higher disease risk [19].

Rural communities in LMICs are predicted to experience greater combined pathogen hazards, and exposure and vulnerability to these hazards [3,19,20] due to fragile health systems and extreme climate events (e.g., droughts, wildfires, flooding) [21,22]. Further, in these regions, climate change is expected to shift the distribution of disease vectors and the distribution of humans, domestic animals, and biodiverse wildlife communities [23,24] with potential consequences for local and global health security (e.g., higher risk of emerging infectious diseases (EIDs), greater potential for long-distance pathogen spread, limited ability to detect outbreaks early, and increased burdens of NTDs [25]).

Overcoming these challenges will require understanding the extent to which pathogen hazards are impacted by climate change [26]. Given that impacts of climate change on human infectious disease risk are tightly linked to the impact on animal and **environmental health** [14], this will require a **One Health** approach [27]. Additionally, technological advances (e.g., remote sensing information, real-time data sharing platforms, field-based diagnostic testing and sequencing), disease forecasting, strengthening of health systems, and a wide range of adaptation mechanisms to climate change will be key to helping with strategic transitions. However, there currently is no clear consensus on how to put this into practice in rural LMICs despite the high local and global health threats [25,28]. Consequently, there is a need for a road map on how such an approach can be realistically and sustainably implemented at varying scales.

In this article, we review the implications of climate change for infectious diseases among rural communities in LMICs with a focus on **East Africa**, a hotspot for climate-related hazards (e.g., relatively rapid warming, extreme and long-term drought) [14] and infectious diseases (in particular NTDs) [29,30]. Further, rural communities in this region tend to be especially vulnerable given their limited access to water, sanitation, and health services, dependence on climate for livelihoods, and often marginalization from government priorities [31–33]. Thus, East Africa provides a good example to illustrate how climate change can impact human health and infectious disease risk, and how health systems could transition towards climate-adapted health systems. We describe this transition by presenting key steps that should be taken to better support health services and communities in rural areas in LMICs in the face of climate change. Importantly, the theorical framework put together in the context of East Africa can be applied to many LMICs but would need to be adapted to the local environmental, ecological, socio-economic, and cultural context, which can differ drastically across the world (e.g., the ecological and socio-economic context and infectious diseases present in East Africa as described in Sections 2 and 3 are different from countries in East Asia such as China; [34]).

## 2. Environmental, ecological, and socio-economic impacts of climate change on rural communities in East Africa

The impact of infectious diseases on the health of rural communities in LMICs is influenced by shifts in environmental, ecological, and socio-economic conditions happening locally, nationally, and globally.

### 2A. Environmental and ecological impacts

**Fig 1:**
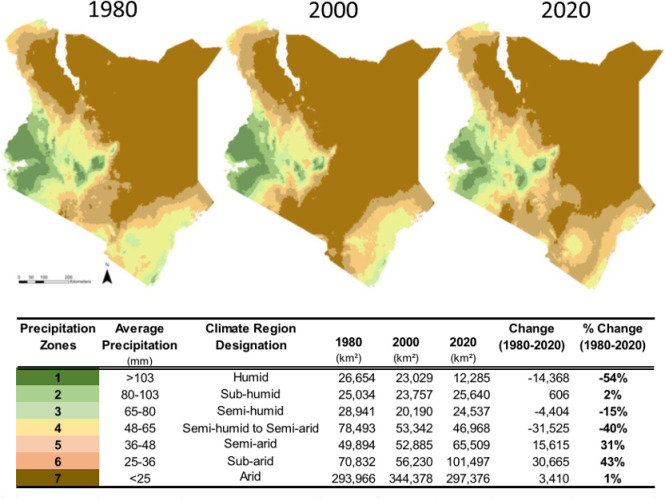
Shift in climate zones across Kenya for the years of 1980, 2000, and 2020 (from Lawrence et al. [40]).

For East Africa, key environmental impacts of climate change include extreme and unpredictable droughts in dryland areas [35,36], flooding in forest and coastal regions [37,38], and other climate hazards [39]. Additionally, drylands, which make up much of East Africa, have shifted from semi-arid to arid landscapes due to frequent droughts [37,40–44], such as in Kenya (Fig 1).

While the consequences of such ecological changes have barely been explored in East African landscapes, theoretical patterns at a global scale suggest that below and above ground microbial communities can be disrupted, along with animal communities such as mammal and bird diversity [1,45,46]. In fact, climate change is a major contributor to African biodiversity loss [47–49], due to habitat change (i.e., loss, alteration, or degradation), wildlife community imbalance, temperature extremes, as well as floods and drought [32,48,50]. Impacts on **ecosystem services** are also apparent (e.g., shrinkage of rangelands altering carbon sequestration [51], proliferation of invasive species [41,52,53]) with important consequences on human and animal health and wellbeing (e.g., loss of food and water provision, productive land, ecotourism [32,47]).

### 2B. Socio-economic impacts

The effect of climate change on the environment and specific ecosystem services has subsequent socio-economic impacts on rural communities. Poor soil fertility, degraded land, limited freshwater availability, and increased presence of agricultural pests with climate extremes have direct effects on crop yield and livestock production [21,22,54]. These losses in agricultural productivity cause food and water insecurity resulting in malnutrition and thus immunocompromised people and animals [21,55–57]. Hampered livelihoods, reduced incomes, physical capital, and capacity to invest have direct social impacts on farming households, limiting their capacity to face other expenditures, such as health and education [58].

For pastoral communities, that contribute 15–60% of meat and milk production in East Africa [59–62], unpredictable precipitation and degraded pastures are affecting migration routes and distances, as well as overlap with wildlife and other herding communities [63–66]. Such environmental stressors push wildlife, domestic animals, and humans into the same or new habitats where there is fertile land and water, thus creating additional competition to already scarce resources, leading to enhanced human-wildlife conflicts and insecurity [67,68]. Such conditions can also lead residents to move away from uncertain climate-sensitive careers with potential consequences on national development priorities.

Climate change impacts on rural households and markets has cascading effects on national economies with disrupted food supply chains, lower quantity and quality of food types, and volatile prices [69–71]. Importantly, while the economic and social effects of climatic extremes, such as the inability to save and invest (e.g., in livestock production, health and wellbeing causing inequality (education, gender) [61]) are well known, feasible solutions to overcome these challenges remain scarce (but see [72,73] for recent innovative solutions to overcome economic impacts of climate change).

## 3. Impacts of climate change on human infectious disease risk in rural areas

To investigate the impacts of climate change on human infectious disease risk, we break down disease risk into 1) pathogen hazard; 2) exposure; and 3) vulnerability to pathogen hazards.

### 3A. Impacts on pathogen hazards—An ecological perspective

Impacts of climate change on the ecology of pathogens can occur both directly and indirectly across multiple spatial scales, filtering down to affect the suitability of ecological niches within which pathogens can survive and reproduce. However, impacts of climate change on pathogen transmission and maintenance will vary with pathogen transmission mode (e.g., environmentally-transmitted, vector-borne, directly-transmitted pathogens), host immunity, and co-infection dynamics. For instance, climate extremes observed in East Africa are having direct impacts on environmentally-transmitted pathogens (i.e., water-borne, food-borne, and soil-transmitted parasites and pathogens) [74,75] (Table 1), with key examples being observed across many LMICs include *Vibrio cholerae*, *Salmonella*, *Campylobacter*, and *Escherichia coli* sp. [76,77].

**Table 1:** Examples of how climate change can impact human, domestic animal, and/or wildlife health, with a focus on infectious diseases in rural areas in East Africa and under the hazard, exposure, vulnerability framework of infectious disease risk (with the hazard being the pathogen that causes harm to human health).

Pathogen hazard	Climate change-related hazard(s)	Human exposure	Human vulnerability	Reference
Middle East respiratory syndrome coronavirus	aridification	Greater exposure to camel-borne diseases with transition from cattle-based to camel-based farming system (farmer exposure but also through the value chain with shifts in meat and milk demands)	Weak access to healthcare	[100]
*Schistosoma* sp.	Warm, stagnant waters	Exposure to parasitic worms and snails due to more favorable water conditions for snail and parasite survival and reproduction	Low access to healthcare	[101]
*Plasmodium* sp.	Severe flooding in the Ugandan highlands	Novel human contacts with malaria mosquitoes	Little protection or awareness of new vector	[102]
*Vibrio cholerae*	Extreme flooding	Greater exposure to disease with stagnant contaminated water	Limited access to healthcare and low surveillance	[75]
*Bacillus anthracis*	Extended periods of drought and/or heavy rainfall	Greater spatial-temporal overlap with wildlife around scarce pastures and water points	Little access to healthcare and low surveillance	[103,104]

Vector-borne pathogens are also of increasing concern in East Africa with warming trends. Micro-climatological factors (e.g., surface water temperatures, soil moisture) that favor rates of development and multiplication of arthropod vectors (either directly or through habitat suitability) can increase prevalence and spread of vector-borne diseases to new regions. For example, in East Africa, Rift Valley Fever virus is expected to become more widespread as its disease-competent vector increases its range under future climate scenarios [78]. Similarly, modelling of temperature-based-traits of mosquitoes and malaria parasites suggest an additional 75.9 million people are expected to be at risk of exposure to malaria by 2080 in Eastern and Southern Africa alone [79]. Other notable examples of vector-borne pathogens that are expanding into new areas of East Africa and other LMICs under climate change include Dengue virus, *Leishmania* spp., West Nile virus, and Zika virus [76,77,80,81]. Thus, there is a pressing need to develop high resolution tools that can help health authorities know when and how to respond to climate change-driven disease threats.

Changes in the geographical range of wildlife hosts in response to climatic shifts will also lead to more opportunities for transmission of directly-transmitted pathogens, including transmission to humans. The magnitude of risk posed to human health by the **ecological release** of novel pathogens from wildlife populations will be informed by opportunities for transmission across interfaces between humans, domestic animals, and wildlife. This in itself is likely to be influenced by climate change. For instance, periods of food insecurity associated with drought can lead to increased bushmeat consumption [82]. Similarly, in dryland areas of sub-Saharan Africa where water is a focal resource that attracts wildlife, livestock and people, low rainfall can cause certain wildlife and livestock species to congregate more strongly at water sources, increasing the potential for cross-species transmission and novel pathogens to emerge [83].

Nutritional deficiency and stress may render individuals more susceptible to infections, possibly causing large outbreaks of known and novel infectious diseases. Additionally, the impact of nutritional deficiency and stress on host immune response with climate change are also likely to impact co-infection dynamics, with downstream consequences that include **synzootics** and **syndemics** in animal and human populations, respectively [84,85]. For example, periods of extreme drought followed by heavy rainfall in East Africa have brought about concomitant epidemics of canine distemper virus and tick-borne babesiosis, resulting in unprecedented mortality in wild carnivore populations [86]. Studies to further elucidate the mechanisms linking climate change, physiological stress, and co-infection dynamics are urgently needed.

### 3B. Impacts on human exposure to pathogen hazards

Climate change impacts on human exposure to pathogen hazards tend to occur primarily through shifts in human and animal (domestic and wildlife) movement, or through human response to climate change impacts such as agricultural production systems, and individual socio-economic status and activities [87]. For example, shifts in temperature and precipitation can alter the range of certain wildlife species and vectors, increasing human exposure to infectious agents not typically present in a given region, as has been observed for both zoonotic diseases and vector-borne diseases in the case of rodent-borne diseases (e.g., *Leptospira* sp.) and zoonotic diseases in bushmeat hunters [88].

In East Africa, climate-related changes in **transhumance** and migration patterns among mobile populations will push these communities into new environments, where ecological and social conditions could lead to changes in contact with livestock and wildlife. Greater human—domestic animal—wildlife contact due to competition for scarce resources brings a high likelihood of novel pathogen transmission in disease-naive populations in settings where disease detection may be dramatically delayed, increasing the risk of disease outbreaks and amplification. At a larger scale, shifts in food supply chains to adapt to climate change impacts can also increase human exposure to certain pathogens. For example, enhancing plant irrigation systems to withstand challenges associated with droughts has the potential to increase exposure to vector-borne diseases [15]. Thus, human exposure to infectious disease hazards is complex and should be explored at different spatial scales while accounting for different ecological and socio-economic factors.

### 3C. Impacts on human vulnerability to pathogen hazards

Human vulnerability to climate change can be observed at the individual level with greater susceptibility to infection and at the population level with weakening of health systems. Climate change has the potential to increase individual susceptibility to infection through impacts that climate extremes have on other health aspects (e.g., nutrition, mental and physical health). Individuals faced with energetic and nutritional stress - through processes linked to hormone release, chronic inflammation and oxidative stress - can have weakened innate and adaptive immune responses, making them more susceptible to infection [89,90]. Such stressors can occur as a result of malnutrition and exposure to heat extremes - factors that humans, domestic animals, and wildlife are increasingly likely to encounter at a global scale [91–94].

Fragile health systems add to the vulnerability in East Africa. Besides new infections in immune naive populations, climate change-related health impacts could exacerbate existing disease burdens in human and animal populations, stressing healthcare delivery. As such, health systems need to develop climate adaptation approaches to withstand climate change impacts, and national strategic plans for endemic diseases may need to be revised based on anticipated ecological and economic impacts of climate change.

Populations that already have limited access to healthcare, in particular transhumance populations and other marginalized communities [33,95,96] will likely be further marginalized with climate change-disrupted healthcare systems. Immunization campaigns and already under resourced primary care services are likely to be disrupted, leading to gaps in coverage among some of the highest-risk communities. Increased human migration due to climate impacts and/or conflict means that overburdened health systems need to be further supported to cope with the influx of **climate refugees** [97]. Therefore, climate change creates new challenges for healthcare systems, humanitarian access, and supply chains where these logistics are already a major challenge due to factors including insecurity, inadequate resources, and inaccessible terrain [98]. Food insecurity and malnutrition are exacerbated in settings of protracted conflict, and will be compounded by the disruptions posed by climate change, leading to increased susceptibility of nutritionally-stressed populations [99].

## 4. Are current health systems prepared for climate change impacts on health?

Given current global climate change predictions, health systems need to be prepared for increasing burdens of infectious diseases, new infectious disease scenarios, with repercussions on other aspects of human and animal health (e.g., nutritional deficiency, mental and physical stress) and economies (e.g., shifts in food availability and prices, employment possibilities). Human infectious disease risk has the potential to increase in rural East Africa because of increased pathogen hazards, exposure, and vulnerability with climate extremes.

Current health systems use traditional disease surveillance and control approaches that are based on passive detection and response to disease outbreaks [105,106]. Such an approach can suffer from underreporting and a biased representation of diseases in the community, with often greater representation of urban than rural populations [107,108]. This is particularly concerning in rural East Africa where there tends to be a disconnect between health services and communities because of socio-economic, cultural, and awareness factors, and in some cases insecurity, lack of infrastructure and human resources. These gaps in public health security combined with minimal understanding of the ecological impacts of climate change in these landscapes may cause late detection of disease outbreaks as well as emergence of novel diseases.

Strengthened and proactive disease surveillance and control that uses high resolution tools and is based on inter-sectoral, One Health partnerships and predictive ecological and epidemiological modelling will allow for cost-effective, targeted interventions [26]. Training of health professionals (i.e., medical doctors, nurses, veterinarians, community health workers) to monitor and treat infectious diseases in the context of a changing climate that impacts different aspects of infectious disease risk (i.e., pathogen hazard, exposure, and vulnerability) is also a fundamental step to take in the transition towards climate-adapted health systems. Health system preparedness and resilience to future climate change and associated health scenarios has been put into question globally [109,110], including in LMICs (e.g., Ghana, Kenya, Nigeria, South Africa, and Tanzania; [111–113]), suggesting that a framework on how health systems should transition towards climate change-resilient health systems is timely. In our proposed framework, we highlight the need for health systems to integrate climate change impacts by exploring the three compartments of infectious disease risk: pathogen hazard, exposure, and vulnerability.

## 5. Steps to strengthen infectious disease prevention, control, and treatment in rural areas in LMICs in the face of climate change with a focus on East Africa

**Fig 2:**
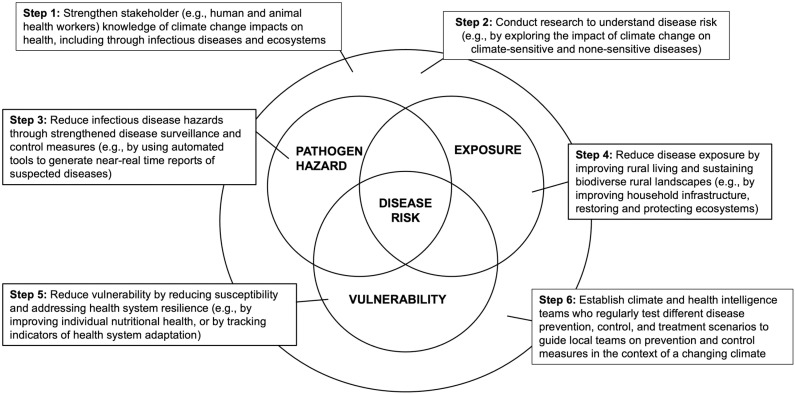
Conceptual diagram illustrating how the 6 steps fall into the pathogen hazard—exposure—vulnerability framework.

In this section, we present six steps that will allow health systems to transition from traditional approaches to ones that build climate adapted and resilient health systems (Fig 2).

### Step 1—Strengthen stakeholder knowledge of climate change impacts on health

Stakeholders involved in the management of infectious diseases (i.e., human and animal health workers, veterinarians, community representatives, ecologists, epidemiologists, physicians, health decision-makers, and intergovernmental and government officials) must understand impacts of climate change at a regional scale, on the environment, economies, infectious diseases, and on health systems. The concept of infectious disease risk being a product of pathogen hazard, exposure, and vulnerability, as well as One Health and **Planetary Health** concepts must also be adopted. Most LMICs already have One Health workforces in place and empowering teams to incorporate a climate change angle to health will be an important next step.

### Step 2—Support research to better understand human infectious disease risks in rural areas

After stakeholder trainings, with emphasis on strong interdisciplinary and multi-sectoral teams, climate change impacts on pathogen hazard, exposure, and vulnerability need to be quantified. Research that can help understand differences in pathogen hazards with climate change includes firstly to empirically investigate the occurrence, distribution, and likely shifts in climate-sensitive pathogens and vectors (i.e., food-, water-, and vector-borne diseases). This can be done by combining national and departmental/county health data with climate data to understand how climate impacts affect disease outbreaks locally (while accounting for the time lag between climate impacts and disease outbreaks). Secondly, the impacts of climate change on non-climate sensitive zoonotic pathogens must be investigated, through shifts in host distributions (e.g., impact on migration routes for humans and livestock as well as wildlife corridors).

Better understanding of human exposure to pathogen hazards can be achieved by firstly exploring dynamics of infectious diseases under different socio-economic and environmental contexts. Combining satellite imagery, meteorological, and ecological and socio-economic models can help facilitate these efforts [114], leading to predictive models that can be turned into decision support tools. Additionally, the timing of interventions must be determined (e.g., annual timing of vaccination campaigns), which will first require understanding the distribution of humans, domestic animals, and wildlife in rural areas and variation with climate (e.g., obtain more robust estimates of the number and distribution of susceptible hosts in rural areas).

Secondly, differences in human and animal exposure to pathogen hazards should be explored at a broader scale, specifically in the context of other global climate change shifts. For example, global market shifts to mitigate climate change impacts, such as meat consumption and production is influencing the distribution and trade network of livestock globally, possibly causing a reshuffling of epidemic risks in certain countries. Given that global food production is associated with greater zoonotic disease risk [115], and global livestock trade can increase the spread of infectious diseases (e.g., Rift Valley fever, African swine fever; [116,117]), understanding how climate mitigation strategies will influence these dynamics is an important area of research that requires further investigation. Hence, the need to make predictions of future health exposures under different scenarios must be considered based on local level climate conditions [3] but also large scale global shifts in trade and other economic decision-makings, and how individual exposure will vary [115].

For vulnerability, research priorities should focus on understanding how climate change will impact individual susceptibility to infection. This includes understanding how other climate change health hazards will occur and interact to influence susceptibility to pathogens. Likewise, co-infection dynamics will be essential, particularly with regards to understanding how current infectious diseases in LMICs (e.g., NTD) will influence the distribution of climate-related emerging diseases.

Subsequently, differences in disease risk can be explored based on differences in pathogen hazard, exposure, and vulnerability observed under different environmental, socio-economic, and health provision scenarios. Such analyses will involve developing accurate probabilistic forecasts of disease risk under different climate scenarios and contexts, and track climate-mediated infectious disease burdens so that surveillance, control, and treatment resources can be structured accordingly.

### Step 3—Reduce health impacts from pathogen hazards through strengthened disease surveillance and control measures in human, domestic animal, and wildlife populations

Along with augmenting research and forecasting, detection and characterization of pathogens and associated diseases in human and animal populations must be reinforced in rural locations. An understanding of the ecology and epidemiology of local microbes, pathogens, and parasites can guide these efforts with targeted and cost-effective benefits (e.g., early detection of outbreaks and novel diseases). However, pathogens and associated disease burdens in rural LMICs are often poorly understood because reliable, high-resolution information on the distribution of rural people and their animals remains scarce across much of the African continent. This, coupled with geographic remoteness, often leads to their omission from health surveillance systems.

Technology that improves the detection and reporting of infectious agents in rural communities could help overcome barriers to their participation in such systems. Automated tools that use machine learning to generate near real-time reports of actively inhabited settlements could be used alongside conventional outreach to increase participation in disease surveillance activities and representation in response efforts to address climate-induced disease threats. Technology can also play an important role in positioning diagnostic testing capacity closer to the point of care, by providing more point-of-care diagnostics, and linking rural communities and associated livestock to centralized disease reporting [118,119]. This combined with remote sensing data, used to detect locations with most change would allow for changes in the occurrence and distribution of pathogen hazards with climate change to be monitored in near-real time. For example, integrating cell phone-based syndromic surveillance of humans and animals and field diagnostics into existing surveillance infrastructure would help generate accessible and timely data streams [120]. This will also improve representation of rural LMICs in national and international strategies, transitioning to incorporating climate change impacts on health, such as the World Health Organization (WHO’s) recent infectious disease surveillance approach termed “climate-informed early warning systems” (CI-EWS) [121,122].

For national and international strategies like CI-EWS to be effective in predicting outbreaks of disease under given climate anomalies, an appropriate resolution of data must be prospectively available and accessible, and collected in the same way over time. Such efforts should be accompanied by an open-source database (e.g., Pathogen Harmonized Observatory (PHAROS); [123]) allowing global sharing of climate-sensitive and zoonotic pathogens at the human, domestic animal, and wildlife interface.

### Step 4—Reduce pathogen exposure by improving rural living and sustaining biodiverse rural landscapes

Once human exposure to pathogen hazards has been quantified (Step 2), interventions to reduce exposure should be explored. In the context of rural East Africa this would include developing means to better support communities in climate-impacted rural areas and mitigate disease exposure. Measures that can directly reduce human exposure to pathogen hazards should be made available in culturally acceptable ways for rural communities newly impacted by specific diseases (e.g., bed nets for emerging vector-borne pathogens). Indirect measures to reduce exposure include improving employment and education possibilities allowing rural communities to improve household infrastructure, and therefore reduce pathogen exposure (e.g., less exposure to water-borne pathogens, soil-transmitted helminths).

New climate scenarios may also increase human exposure to wildlife pathogens through greater spatial-temporal overlap of people and animals or human consumption of bushmeat. However, sustaining biodiverse landscapes can in some cases reduce human exposure to certain pathogens through the **dilution effect** [124]. Thus, understanding the human—domestic animal—wildlife interface, and the role of biodiversity in influencing pathogen hazards under different climate scenarios will be essential [125]. Leveraging global networks of digitally available data on biodiversity, such as those harbored in natural history museums [126] and publicly available through online aggregators such as the Global Biodiversity Information Facility (GBIF), can provide critical information to model and understand complex interactions between biodiversity and human disease at multiple spatial scales [127,128]. Given that sustaining biodiversity has multiple **health co-benefits** (e.g., rangelands and forests act as carbon sinks, biodiversity can reduce spread of animal and plant diseases and pests) [129], financial support for rural communities to restore and protect ecosystems (e.g., reforestation, land restoration, control of invasive species) is essential. Such investment will have other societal benefits such as increasing household income, female education, youth employment, ameliorate family planning, and increase access to basic services (e.g., healthcare) (15)—with lasting national economic benefits (e.g., international trade, tourism). This, combined with the deployment of smarter technologies to help support basic household and farming needs in rural areas is critical, as well as conducting awareness campaigns so that target populations are aware of the climate impacts and solutions.

### Step 5—Reduce vulnerability by reducing susceptibility and addressing health system resilience

Vulnerability can be reduced by limiting susceptibility to infection at the individual level and making health systems more resilient at the population level. Susceptibility to infection can be reduced by improving nutritional health and through vaccine delivery for infectious diseases predicted to increase with climate change. Supporting rural farmers in accessing and using drought-resistant crops and animals, and genetically diverse agricultural systems (livestock and crops) can make agricultural production systems more resilient to extreme climate events and less prone to disease outbreaks.

Similarly, access to vaccines is essential and yet vaccine inequity and access to healthcare continue to be a global issue, with African countries, especially rural African communities, often most severely impacted [130]. WHO and the World Organization for Animal Health (WOAH) are increasingly facilitating provisions and coordinated responses for LMICs transitioning towards strengthened health systems (e.g., rabies and pest des petits ruminants), and identifying populations most vulnerable to infectious diseases with increasing climate change impacts is an important aspect to integrate into national strategic plans.

Vulnerability can be reduced at the population level by creating climate resilient health systems that can anticipate, respond, and adapt to stressors imposed by climate change [3,131,132]. Such approaches include tracking indicators of adaption, such as adaption of communities and health systems to extreme climate conditions and shifts in the burden of climate-sensitive diseases. This will require conducting Monitoring and Evaluations (M&E) that are specifically directed towards assessing impacts of climate change. For example, comparing differences in patient outcome, quality of care, waiting time, vaccine and antibiotic availability between drought and none-drought periods [133,134].

Transformation of health systems to be climate resilient must include a restructuring of healthcare practices, with an emphasis on training health workers to have adaptable skills and to think more broadly about infectious diseases to adapt to different disease scenarios with climate change [25]. Currently, disease diagnosis and patient care typically focus on well-known diseases (e.g., fever-like syndromes diagnosed as malaria instead of exploration of potential novel zoonotic diseases [135]) which may lead to misdiagnosis if infectious diseases are reshuffling with climate change. Hence, health professionals require broader diagnostic thinking with climate change [136,137], which can be facilitated through trainings done by groups such as the Africa One Health University Network (AFROHUN).

### Step 6—Establish climate and health intelligence teams to ensure regular updates in health and climate local and national guidelines

The final step that ties Steps 2–5 together by touching on all aspects of infectious disease risk (i.e., pathogen hazard, exposure, and vulnerability; Fig 2) is the need to establish climate and health intelligence teams located at the county/departmental and national levels. The role of these teams is to regularly test different disease prevention, control, and treatment scenarios based on current and predicted future climatic shifts, and then inform local teams on how to adapt surveillance, control, and treatment measures. This will require effective communication and data sharing across sectors and spatial scales, including internationally to obtain global estimates of climate shifts. These teams would also be tasked to evaluate shifts in socio-economic factors at a national and global scale, which could have indirect effects on infectious diseases at different spatial scales, including in rural areas in LMICs. Such a climate and health intelligence system will help reduce pathogen hazards, exposure, and vulnerability, as well as move away from traditional ‘reactive’ responses to disease outbreaks and towards ‘proactive’ responses.

## 6. Conclusions

Climate change impacts on human and animal health are increasingly apparent, and most countries have begun planning and implementing climate change adaptation strategies [14]. Alongside these climate actions, health systems need to transition to climate-resilient health systems so that disease surveillance, control, and patient care are not impacted by climate hazards. The COVID-19 pandemic has pushed health authorities across the world to strengthen disease surveillance and control. Such efforts must now be put in the context of a changing climate so that global health security is maintained and progress towards achieving Sustainable Development Goals is not impacted. Our framework provides guidance on how to successfully transition health systems towards climate adaptation by focusing on communities that are impacted by climate change and frequently marginalized from health systems—rural communities in LMICs. The framework is presented in the context of East Africa and can be adapted to other ecological, socio-economic, and cultures contexts.

## Supporting information

S1 Text Glossary of terms. (DOCX)
