# Supplementary material for: A framework for managing infectious diseases in rural areas in low- and middle-income countries in the face of climate change—East Africa as a case study
Source: PLOS Glob Public Health. 2025 Jan 30;5(1):e0003892. doi: 10.1371/journal.pgph.0003892 (PMC11781624; doi:10.1371/journal.pgph.0003892)
Supplement: S1 Text — (DOCX) [file pgph.0003892.s001.docx]

**Manuscript title:** A framework for managing infectious diseases in rural areas in low- and middle-income countries in the face of climate change – East Africa as a case study

**SUPPORTING INFORMATION**

**S1 Text:**

**GLOSSARY OF TERMS**

**Climate change:** Long-term shifts in temperature and weather patterns.  **Climate sensitive diseases:** Diseases influenced by changes in temperature or weather conditions.
**Climate refugee:** Individuals that must leave their homes due to climate change impacts. **Dilution effect:** Circumstance in which the diversity of vertebrate hosts present in a community reduces pathogen transmission [1]. **Disease risk:** The likelihood of disease caused by the product of the pathogen hazard, and exposure and vulnerability to the pathogen hazard [2].

**East Africa:** Countries include Burundi, Djibouti, Eritrea, Ethiopia, Kenya, Malawi, Mozambique, Rwanda, Somalia, South Sudan, Tanzania, and Uganda.

**Ecological release:** Population increase that occurs when a species is released from constraints in its environment. **Ecosystem services:** Ecological processes and functions which provide benefits to individuals or society [3].

**Environmental health:** Field focused on understanding how aspects of the environment affect human health. **Exposure:** Human exposure to a disease hazard. **Health co-benefits:** Climate change mitigation actions that benefit human health through different angles (e.g., lower risk of infectious diseases and lower exposure to pollutions).

**Health hazard:** A factor that is a potential source of harm to health [4].

**One Health:** Term used to acknowledge the interconnectedness of human, animal, and environmental health and the need to study all three simultaneously.

**Pathogen hazard:** An infectious agent that has the potential to cause harm to health [2,4]

**Planetary Health:** Area of work focused on understanding the interconnectedness of human health with natural systems [5].

**Syndemics:** When two or more diseases or health conditions are co-present causing worse health outcomes [6].

**Synzootics:** Occurrence of two or more diseases within an animal population causing worst health outcomes at the population level than if the diseases occurred separately [7]**.
Transhumance:** Periodic migration of livestock based on pasture availability. Movement done in a seasonal fashion.

**Vulnerability:** At the individual level, higher disease risk due to higher susceptibility to infection, and at the population level due to, for example, limited access to healthcare **Wildlife reservoir:** Wildlife population or species that can maintain an infectious agent independently without external input (e.g., introduction or spillover from other populations or species).

**REFERENCES**

1. Ostfeld RS, Keesing F. Biodiversity series: The function of biodiversity in the ecology of vector-borne zoonotic diseases. Can J Zool. 2000;78: 2061–2078. doi:10.1139/z00-172

2. Gibb R, Franklinos LHV, Redding DW, Jones KE. Ecosystem perspectives are needed to manage zoonotic risks in a changing climate. BMJ. 2020; m3389. doi:10.1136/bmj.m3389

3. Pörtner H-O, Scholes RJ, Agard J, Leemans R, Archer E, Bai X, et al. IPBES-IPCC co-sponsored workshop report on biodiversity and climate change. 2021; doi:10.5281/zenodo.5101133

4. Hosseini PR, Mills JN, Prieur-Richard A-H, Ezenwa VO, Bailly X, Rizzoli A, et al. Does the impact of biodiversity differ between emerging and endemic pathogens? The need to separate the concepts of hazard and risk. Philos Trans R Soc B Biol Sci. 2017;372: 20160129. doi:10.1098/rstb.2016.0129

5. Whitmee S, Haines A, Beyrer C, Boltz F, Capon AG, Dias BF de S, et al. Safeguarding human health in the Anthropocene epoch: report of The Rockefeller Foundation–Lancet Commission on planetary health. The Lancet. 2015;386: 1973–2028. doi:10.1016/S0140-6736(15)60901-1

6. Singer M, Bulled N, Ostrach B, Mendenhall E. Syndemics and the biosocial conception of health. The Lancet. 2017;389: 941–950. doi:10.1016/S0140-6736(17)30003-X

7. Sweeny AR, Albery GF, Becker DJ, Eskew EA, Carlson CJ. Synzootics. J Anim Ecol. 2021;90: 2744–2754. doi:10.1111/1365-2656.13595
